# Complex port removal: balloon-assisted retrieval of retained intravascular catheters – a technical note

**DOI:** 10.1186/s42155-025-00589-0

**Published:** 2025-10-16

**Authors:** Nicholas L. Thomas, Coplen D. Johnson, Christopher Stevens, Joseph Eskew, Chiranjiv Virk, Chaitanya Ahuja, Paul E. Perkowski, Heba Fouad

**Affiliations:** 1 Department of Interventional Radiology, Louisiana State University Health Sciences Center Shreveport, 1501 Kings Highway, Shreveport, LA 71103 USA; 2 Department of Vascular Surgery, Louisiana State University Health Sciences Center Shreveport, 1501 Kings Highway, Shreveport, LA 71103 USA

**Keywords:** Balloon-assisted retrieval, Port-a-cath, Central venous catheter, Retained catheter, Case series

## Abstract

**Purpose:**

Balloon dilatation has remained relatively underutilized despite its emerging potential for hemodialysis catheter removal. This case series revisits balloon-assisted retrieval to showcase its effectiveness in retrieving long-term port-a-caths complicated by fibrotic encapsulation, calcification, and stenosis.

**Materials & methods:**

Endoluminal balloon-assisted retrieval was utilized in 12 cases with retained port-a-cath catheters after gentle traction failed to remove the indwelling 8 French catheter due to complex intravascular fibrotic adhesions and calcifications. Under fluoroscopic guidance, a non-compliant angio-balloon was advanced over a guidewire through the externally accessible central venous catheter. Once the balloon reached the distal segment of the tubing, it was sequentially inflated in a retrograde manner within the catheter's lumen to disrupt the surrounding adhesions.

**Results:**

Balloon-assisted retrieval was successfully performed in all 12 cases, allowing for the complete removal of the indwelling catheter via gentle traction despite significant fibrotic encapsulation, calcification, and stenosis. Minimal blood loss was observed, and no further complications were reported.

**Conclusion:**

This case series revisits endoluminal balloon dilatation, an emerging technique for removing hemodialysis catheters, to demonstrate its application and efficacy in retrieving long-term port-a-cath catheters complicated by fibrotic encapsulation, calcification, and stenosis. This technique should be regarded as a primary retrieval option for retained port-a-caths in instances of severe fibrosis and calcification.

**Level of evidence:**

Level 4, case series.

## Introduction

Central venous catheterization (CVC), such as the port-a-cath, is a common method of vascular access for chronically ill patients requiring long-term therapy. However, prolonged catheterization increases the risks of infection, catheter/vessel thrombosis, and stenosis due to fibrin sheath formation [1]. Therefore, prompt removal of long-term catheterization is necessary once it is no longer needed.

While gentle catheter traction remains the standard removal technique, long-term catheterization with adhesions and/or calcifications can complicate this process, often necessitating an advanced procedure [1]. In instances where catheter removal is complicated, open surgical removal was previously warranted, as forceful catheter removal could result in fragmentation, embolization, and potentially life-threatening vessel and atrial tearing [2]. However, in 2011, endoluminal balloon dilatation was introduced as a method to free fibrosed hemodialysis catheters from their endovascular adhesions [3]. This innovative technique enabled the safe and effective retrieval of retained catheters with complex adhesions and calcifications [3, 4].

Inspired by this approach, we implemented endoluminal balloon-assisted retrieval as the primary technique for extracting externally accessible retained port catheters. This manuscript adds to the expanding documentation supporting this technique and emphasizes its potential as a primary method for removing retained port-a-caths, particularly in cases involving complex fibrin sheath formations and calcifications.

## Materials & methods

From January 2019 to October 2024, twelve patients with long-term port-a-caths underwent endoluminal balloon-assisted retrieval (Table 1). All were diagnosed with retained catheters during port-a-cath removal after gentle traction failed to retrieve the CVC tubing from the vascular tree. All retained catheters were externally accessible, which reduced costs, risks, procedural steps, and time to completion, as we did not require additional percutaneous access. Three cases were identified at an outside hospital after the port was removed via blunt dissection and were subsequently referred to our hospital.

**Table 1:** Patient case and central venous catheter details upon presentation

Case	Patient Age	Patient Sex	Reason for Catheterization	Type of Catheter	Side of Port	Age of Catheter	Port Present^a^
Case 1	56	F	Lupus and Scleroderma	Port-a-Cath	Right	15 years	No
Case 2	73	F	Right-Sided Breast Cancer	Port-a-Cath	Left	8 years	No
Case 3	61	F	Lupus and Rheumatoid Arthritis	Port-a-Cath	Left	12 years	No
Case 4	61	F	Metastatic NSCLC Adenocarcinoma	Port-a-Cath	Right	11 years	Yes
Case 5	55	F	Sickle Cell Disease	Port-a-Cath	Left	16 years	Yes
Case 6	44	M	Sickle Cell Disease	Port-a-Cath	Left	14 years	Yes
Case 7	43	F	Sickle Cell Disease	Port-a-Cath	Right	10 years	Yes
Case 8	71	F	Bilateral Breast Cancer	Port-a-Cath	Left	14 years	Yes
Case 9	40	M	Non-Hodgkin’s Lymphoma	Port-a-Cath	Left	9 years	Yes
Case 10	21	F	Gaucher’s Disease Type 3	Port-a-Cath	Right	18 years	Yes
Case 11	66	M	Gastric Lymphoma	Port-a-Cath	Right	29 years	Yes
Case 12	49	F	Calcium Gluconate Infusions	Port-a-Cath	Left	15 years	Yes

^a^Some ports were removed at an outside hospital and pericatheter adhesions were identified at that time

**Fig. 1:**
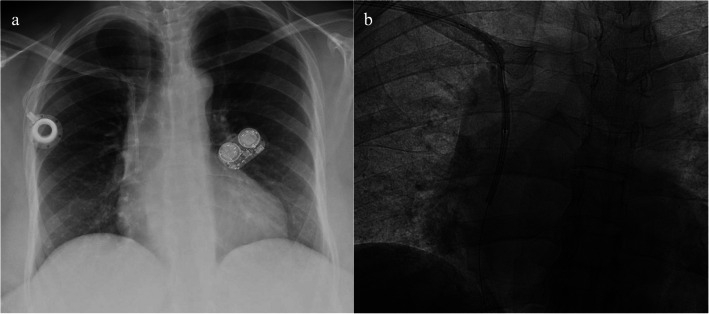
**a**: preoperative PA chest x-ray demonstrating right-sided chest port with a subclavian tunneled catheter and pericatheter calcifications along its course through the superior vena cava; **b**: intraoperative fluoroscopy demonstrating angioplasty with a 3 mm balloon as it disrupts the calcified adhesions to allow for removal of the catheter in its entirety without fragmentation

Upon arrival at the angio-suite and under sterile conditions, we performed a preliminary fluoroscopic evaluation to visualize the retained catheter and surrounding adhesions in the vascular tree (Fig. 1a). We would then attempt removal of the catheter via manual pulling to confirm the necessity for balloon-assisted retrieval. After identifying the retained catheter, we advanced a guidewire into the lumen of the external portion of the catheter tubing. We then advanced a non-compliant balloon over the wire, entering the lumen until we reached the distal end of the catheter tubing, past the identified adhesions, as confirmed by fluoroscopy (Fig. 1b).

**Fig. 2:**
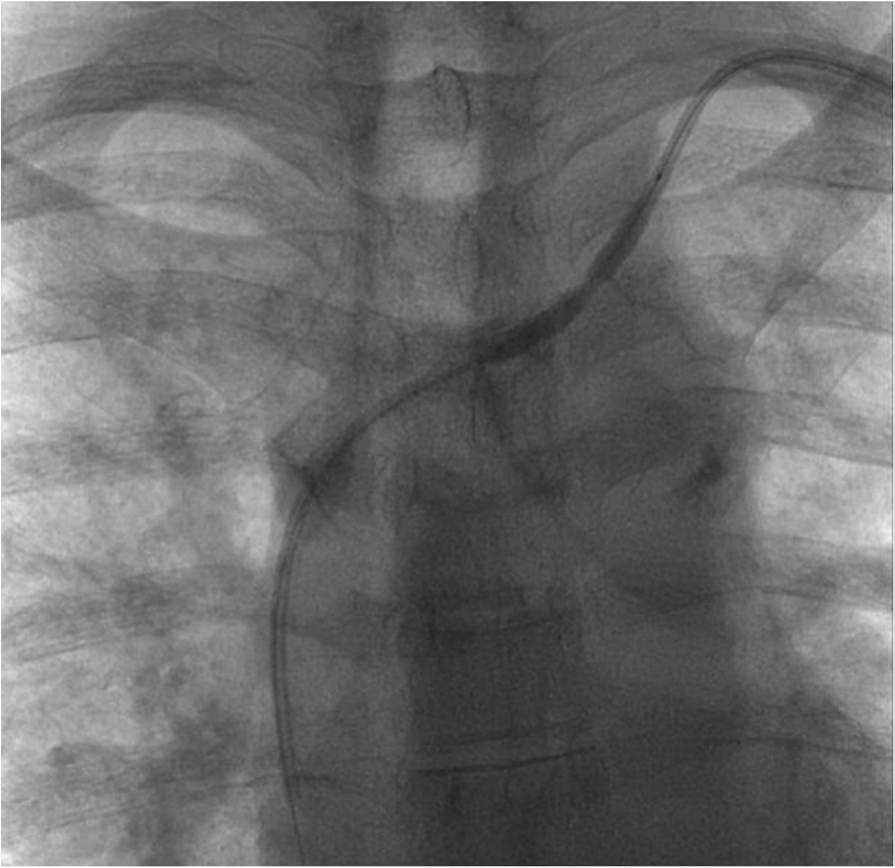
Intraoperative fluoroscopy demonstrating angioplasty of a left-sided central venous catheter with adhesions to the vessel wall of the left brachiocephalic and internal jugular vein; a waist is noted in the midportion of the balloon, demonstrating a segment of stenosis secondary to pericatheter adhesions

**Fig. 3:**
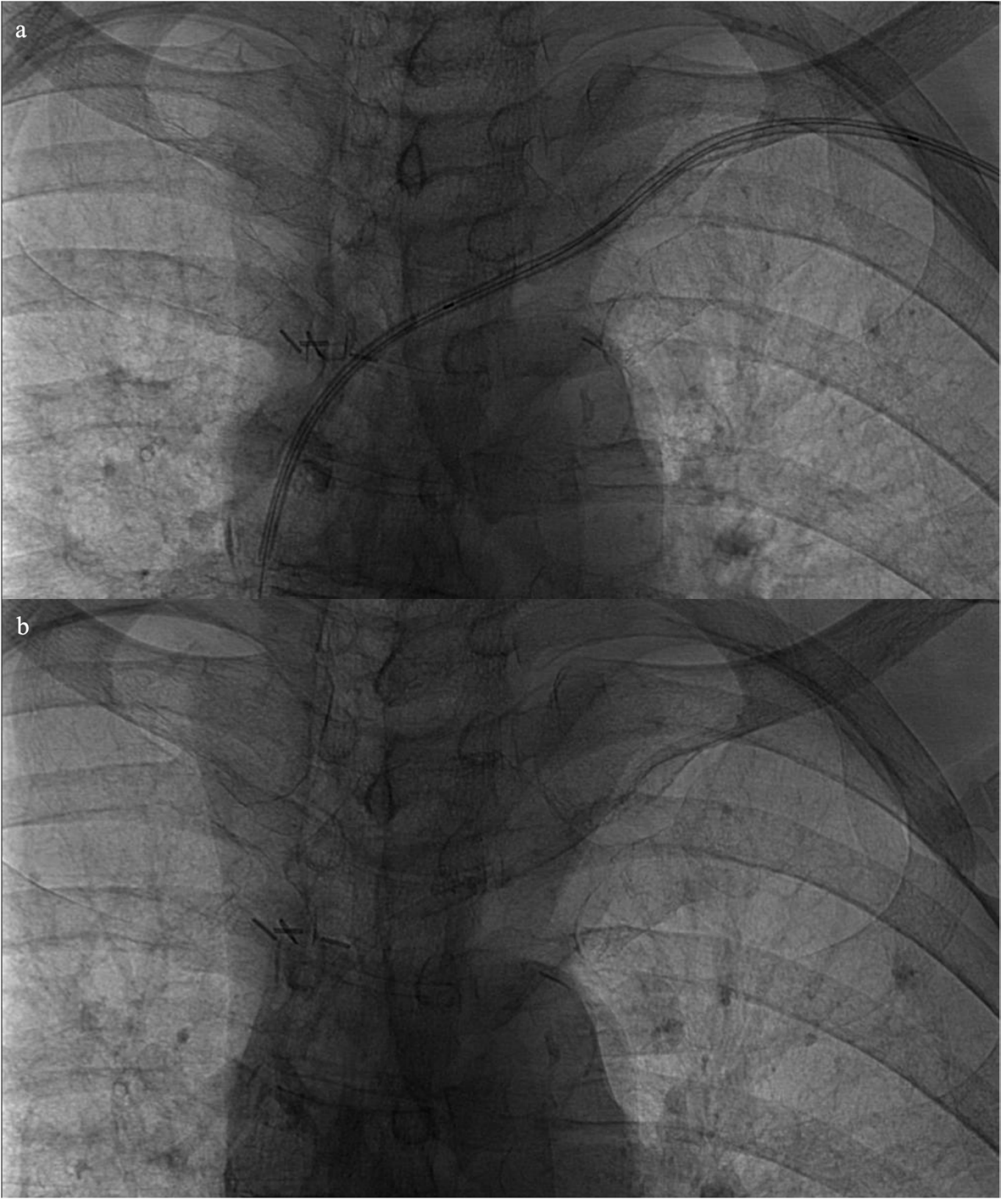
**a** Intraoperative fluoroscopy during angioplasty of a left-sided port-a-cath demonstrating the radio-opaque markers used to visualize the edges of the balloon; **b** postoperative fluoroscopy demonstrating the residual calcifications along the walls of the left innominate vein after the removal of the catheter in its entirety

Once we visualized the balloon at the distal end of the retained catheter, we performed serial dilatation and deflation from the distal end back to the external end of the tubing (Fig. 2). Because of their increased endoluminal surface area, longer-length balloons required fewer dilatations to expand the entire length of the tubing, while shorter-length balloons required more dilatations. After disrupting the fibrotic encapsulations and calcifications, we could safely extract the catheter in its entirety with gentle traction (Fig. 3). Our institution has shifted preference towards longer balloons for this reason; however, it still depends on the availability of balloons in the procedural suite.

When comparing the technical aspects of the cases, we utilized ten different non-compliant balloons and two types of guidewires (Table 2). The lengths of the balloons and sizes of the guidewires were determined by what was readily available in the procedure suite or by physician preference. The balloon diameter was determined by the estimated size needed to disrupt surrounding adhesions and calcifications. The largest balloon in this series was 6.0 mm, which was used after a 5.0 mm failed to release the catheter from the surrounding adhesions. The initial difficulty in this case may have been due to the port being used as a long-term access site for calcium gluconate infusions, leading to extensive calcifications. Operators at our institution typically use rated pressure (between 12 and 16 mmHg). If the catheter is not freed at the rated burst pressure with one or two dilations, especially at the site of venous insertion, we then increase the size of the balloon. All retained CVCs included in this report were 8 French catheters and were successfully removed in around 10 min from the start of the procedure.

**Table 2:** Procedural and technical aspects of balloon-assisted retrieval cases

Case	Age of Catheter	Reason for Catheter Removal	Guidewire Used for Removal^a^	Balloon Used for Removal^b^	Procedural Complication	Day of Discharge
Case 1	15 years	No longer functioning, recurrent bacteremia	0.014-inch guidewire	3.0 mm x 10 cm Sterling SL	None	Same Day
Case 2	8 years	Completion of treatment	0.014-inch guidewire	2.5 mm x 10 cm Sterling SL	None	Same Day
Case 3	12 years	Recurrent bacteremia	0.014-inch guidewire	4 mm x 22 cm Coyote	None	Post-Op Day 3
Case 4	11 years	Completion of treatment	0.018-inch guidewire	4.0 mm x 4 cm Sterling	None	Same Day
Case 5	16 years	No longer functioning	0.014-inch guidewire	3.0 mm x 4 cm Coyote ES	None	Same Day
Case 6	14 years	No longer functioning	0.014-inch guidewire	4.0 mm x 4 cm Sterling	None	Same Day
Case 7	10 years	No longer functioning, recurrent bacteremia	0.014-inch guidewire	4.0 mm x 22 cm Coyote	None	Post-Op Day 1
Case 8	14 years	Completion of treatment	0.014-inch guidewire	2 mm x 15 cm Coyote	None	Same Day
Case 9	9 years	Completion of treatment	0.014-inch guidewire	3 mm x 15 cm Coyote	None	Same Day
Case 10	18 years	Requested oral treatment	0.014-inch guidewire	2.5 mm x 15 cm Coyote	None	Same Day
Case 11	29 years	Completion of treatment	0.014-inch guidewire	4.0 mm x 15 cm Coyote	None	Same Day
Case 12	15 years	Recurrent bacteremia	0.014-inch guidewire	6.0 mm x 22 cm Sterling	None	Post-Op Day 7

^a^The size of the guidewire was determined based on physician preference, and what was readily available in the procedural suite

^b^The size of the balloon was determined based on physician preference, preoperative imaging, and trial and error if initial balloon failed to free the catheter tubing from extensive adhesions and calcifications

## Results

The youngest patient in this series was 21 years old, while the oldest was 77, with an average age of 53.3 years. The shortest duration of indwelling catheter time in this series was 8 years, while the longest was 29 years, providing an average catheter indwelling time of 14.3 years. Nine patients were female (75.0%), and seven cases involved left-sided port-a-caths (58.3%).

All procedures were completed successfully, achieving a 100% success rate, minimal blood loss (< 10 mL, without need for pressure bandaging), and no procedural complications. Nine patients were discharged from the hospital on the same day as their procedure; however, three required additional treatment for ongoing bacteremia before discharge.

Recurrent bacteremia was reported as an indication for removal in four cases (33.3%), while a non-functional port-a-cath was indicated in four cases (33.3%). Completion of treatment without further need for indwelling CVC was cited as a reason for removal in five cases (41.7%). Additionally, one patient opted to receive oral medications after being treated via pediatric port-a-cath for 18 years.

## Discussion

**Fig. 4:**
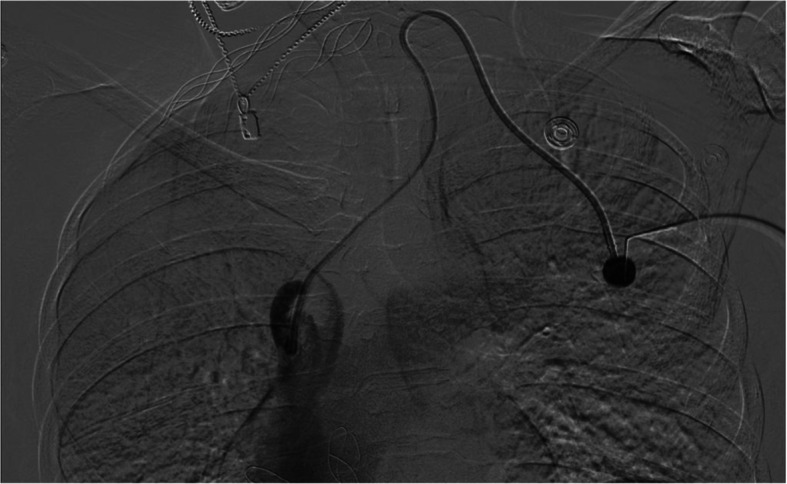
Digital subtraction angiography via a left-sided port-a-cath demonstrating a filling defect and contrast jet at the catheter tip typical of a fibrin sheath formation and the appearance of intravascular calcifications along the distal catheter tip

Prompt CVC removal via manual pulling is the optimal strategy to prevent complications when access is no longer needed. However, in cases of pericatheter fibrotic encapsulation, stenosis, or calcification, we recommend balloon-assisted retrieval as the primary method, especially for retained port-a-caths (Fig. 4), where current standard endovascular techniques have been known to fail [2, 5]. Additionally, it’s important to note that all retained CVCs were externally accessible in this series, allowing us to enter the catheter tubing directly without requiring additional percutaneous access or surgical incisions.

This study's limitations include its retrospective design, small sample size, absence of a control group, variability in balloon and guidewire selection, lack of long-term follow-up, and potential undetected complications (such as vessel injury and thrombosis). Furthermore, it is important to acknowledge that the findings are based on procedures performed by multiple physicians at a single institution, which may not reflect variations in practice, patient populations, or operator experience at other centers.

We opted for balloon-assisted retrieval due to the initial challenges faced during port-a-cath removal and our interest in its reported ease of use, effectiveness, and safety in hemodialysis catheter retrieval [3–5]. Throughout the procedures, we noted that the balloon's ability to disrupt adhesions and safely remove the retained catheter was remarkable, especially in cases where the catheter was firmly adhered or where conventional methods posed a greater risk of vessel injury [2, 5–7]. All cases were successfully completed using balloon-assisted retrieval without complications, consistent with existing data on hemodialysis catheter retrieval, indicating a higher success rate and a lower complication rate than other retrieval methods [5].

Although several well-documented foreign body retrieval options exist, including various endovascular devices and surgical interventions [5], we believe balloon-assisted retrieval provides a more controlled and less traumatic alternative. Furthermore, minimizing cost, risk, and procedure time aligns with our practice's goals of patient-centered care, as current standards for foreign body retrieval often require additional percutaneous access [6, 7], multiple catheters [5–7], additional procedural steps [6, 7], increased risk to patients [5], less delicate instruments [5–7], and longer time to completion. As the use of indwelling CVCs continues to rise, complications such as fibrotic encapsulation, calcification, and stenosis are likely to increase as well. Recognizing pericatheter adhesions and employing appropriate endovascular retrieval will be crucial for effectively managing our aging population.

## Conclusion

Endoluminal balloon-assisted retrieval, an emerging technique for hemodialysis catheter removal, is a safe and effective method for retrieving long-term port-a-caths complicated by fibrotic encapsulation, calcification, and stenosis. Further prospective studies are needed to optimize and refine this technique; however, this minimally invasive approach helps mitigate risks associated with forceful traction and surgical removal while maintaining vascular integrity. Given its high success rate and low complication profile, balloon-assisted retrieval should be regarded as the primary method in cases where standard techniques fall short.

## Data Availability

All data generated or analyzed during this study are included in this published article [and its supplementary information files].
